# Air Pollution and Childhood Respiratory Allergies in the United States

**DOI:** 10.1289/ehp.11497

**Published:** 2008-09-30

**Authors:** Jennifer D. Parker, Lara J. Akinbami, Tracey J. Woodruff

**Affiliations:** 1 National Center for Health Statistics, Centers for Disease Control and Prevention, Hyattsville, Maryland, USA;; 2 Program on Reproductive Health and the Environment, Department of Obstetrics, Gynecology, and Reproductive Sciences, University of California–San Francisco, San Francisco, California, USA

**Keywords:** allergy, children, hay fever, ozone, particulate matter

## Abstract

**Background:**

Childhood respiratory allergies, which contribute to missed school days and other activity limitations, have increased in recent years, possibly due to environmental factors.

**Objective:**

In this study we examined whether air pollutants are associated with childhood respiratory allergies in the United States.

**Methods:**

For the approximately 70,000 children from the 1999–2005 National Health Interview Survey eligible for this study, we assigned between 40,000 and 60,000 ambient pollution monitoring data from the U.S. Environmental Protection Agency, depending on the pollutant. We used monitors within 20 miles of the child’s residential block group. We used logistic regression models, fit with methods for complex surveys, to examine the associations between the reporting of respiratory allergy or hay fever and annual average exposure to particulate matter ≤ 2.5 μm in diameter (PM_2.5_), PM ≤ 10 μm in diameter, sulfur dioxide, and nitrogen dioxide and summer exposure to ozone, controlling for demographic and geographic factors.

**Results:**

Increased respiratory allergy/hay fever was associated with increased summer O_3_ levels [adjusted odds ratio (AOR) per 10 ppb = 1.20; 95% confidence interval (CI), 1.15–1.26] and increased PM_2.5_ (AOR per 10 μg/m^3^ = 1.23; 95% CI, 1.10–1.38). These associations persisted after stratification by urban–rural status, inclusion of multiple pollutants, and definition of exposures by differing exposure radii. No associations between the other pollutants and the reporting respiratory allergy/hay fever were apparent.

**Conclusions:**

These results provide evidence of adverse health for children living in areas with chronic exposure to higher levels of O_3_ and PM_2.5_ compared with children with lower exposures.

Children in the United States are generally healthy; > 80% are in excellent or very good health according to the 2006 National Health Interview Survey (NHIS) (Bloom and Cohen 2007). However, a significant proportion of children are affected by respiratory conditions, including allergies, which are associated with missed school days and activity limitations (Blaiss 2004). Parent responses to questions in the 2006 NHIS indicate that about 9% of children < 18 years of age had had hay fever in the preceding 12 months, and slightly more had had respiratory allergies (Bloom and Cohen 2007). These estimates are higher than comparable statistics from the 1982 NHIS, where 5.5% of children were reported to have had hay fever or allergic rhinitis in the previous 12 months [National Center for Health Statistics (NCHS) 1985]. Respiratory allergies manifest as a variety of symptoms of varying duration and severity. In a recent review, Blaiss (2004) concluded that childhood allergic rhinitis, or allergies, contributes to increased loss of school days, impaired school performance, and increased mental health disorders from the various allergic symptoms and resulting increased sleep disruptions. Recent data from the NHIS support this conclusion; in a simple cross-tabulation, children with respiratory allergies or hay fever were more likely to miss school than those without these conditions (Parker et al. 2007). A brief review by Simons (1996) describes the impacts of allergies, including effects of medications used to treat allergies, on learning impairment in children.

A possible factor affecting respiratory allergic symptoms is exposure to ambient air pollution, particularly traffic pollutants. Reviews of the effects of air pollution on allergies have concluded that pollutants likely exacerbate effects of allergens among those with existing susceptibility, rather than initiating allergies among people without existing allergies (e.g., Bartra et al. 2007; D’Amato et al. 2005; von Mutius 2000). D’Amato et al. (2005) list possible rationales for the associations among components of air pollution, allergens, and allergic response, including the effects of the pollutants on the potency of the allergens and the increased susceptibility of the subjects via an inflammatory effect on the airway, increased airway reactivity, or increased bronchial responsiveness. Experimental evidence, for example, has shown that exposure to diesel particles may increase allergic response (e.g., Knox et al. 1997; Riedl and Diaz-Sanchez 2005), and a recent review of experimental studies outlines possible pathways for these effects (Riedl 2008). Human experimental studies have shown increased allergic response among predisposed adult subjects directly exposed to ozone at high (Jörres et al. 1996) or moderate but repeated (Holz et al. 2002) levels.

No comprehensive studies have examined associations between allergic symptoms and air pollution among a nationwide sample of U.S. children. From Europe and Asia, increases in indices of air pollution have been found to exacerbate childhood allergic symptoms in some studies (Annesi-Maesano et al. 2007; Hajat et al. 2001; Hwang et al. 2006; Janssen et al. 2003; Krämer et al. 2000; Lee et al. 2003; Morgenstern et al. 2007, 2008; Pénard-Morand et al. 2005; Yu et al. 2005) but not all (Nicolai et al. 2003; Ramadour et al. 2000). Some variation in results can be attributed to differences in case definitions. Many studies examine asthma, upper and lower respiratory infection, or other allergic symptoms, such as food or skin allergies, in addition to allergic rhinitis. The specific pollution exposures studied as well as other place-specific factors, such as local flora, may contribute to the differences in findings; across the United States, for example, pollen varieties and seasonal impact vary (Blaiss 2004). An intriguing study by von Mutius et al. (1998) documented the change in the incidence of seasonal allergic rhinitis after the reunification of Germany. Before reunification, allergies and asthma were less common in East Germany, an area with more industrial pollution, than in West Germany, with more traffic pollution. After reunification, allergies, but not asthma, increased among children in East Germany as East Germany’s living conditions became more westernized, including more traffic. In a recent review, Heinrich and Wichmann (2004) concluded that the evidence linking traffic-related air pollutants and allergic rhinitis is equivocal. As argued by Brunekreef and Sunyer (2003), more studies on air pollution and allergic rhinitis are needed.

The objective of this study was to explore possible relationships between air pollution measures and respiratory allergies in a large nationwide sample of U.S. children. In this study, we used data from the 1999–2005 NHIS that had been geographically linked to annual air monitoring data for several pollutants [particulate matter (PM), O_3_, sulfur dioxide, and nitrogen dioxide] from the U.S. Environmental Protection Agency (U.S. EPA 2008). With both air pollution and allergen levels expected to rise with global climate change (Confalonieri et al. 2007), understanding the potential consequences for children’s well-being is important both now and in the future.

## Materials and Methods

### NHIS

The NHIS is a large nationally representative survey of the civilian noninstitutionalized U.S. population (NCHS 2008c). Very briefly, the NHIS is a cross-sectional household interview survey conducted continuously throughout the year. For survey years 1999–2005, after state-level stratification, the first stage of its multistage probability design consisted of a sample of 358 primary sampling units (PSUs) drawn from approximately 1,900 geographically defined PSUs. PSUs are counties or groups of counties, or a metropolitan statistical area. Within a PSU, second-stage units are drawn (segments), and within each segment, all occupied households are targeted for interview. Black and Hispanic populations were oversampled during survey years 1999–2005. The probabilities of selection, along with adjustments for nonresponse and poststratification, are reflected in the sample weights (Botman et al. 2000). For additional information, see NCHS (2008b).

In 1999–2005, about 35,000 households were sampled each year, yielding almost 100,000 total respondents each year. In addition to the core family questionnaire each family member answers, within each family with children, a sample child was selected for additional questions on health and health care (Bloom and Cohen 2007). Response rates were generally high. During these data years, information was provided for > 90% of children selected for the sample child questionnaire; multiplied by the sample family response rates of 85–90%, the unconditional response rate for the sample child is about 80%. Special NHIS files geocoded to U.S. Census block and block group have been created and are available only through the NCHS Research Data Center (NCHS 2008d).

A total of 73,198 children 3–17 years of age provided information for the sample child questions in the 1999–2005 NHIS. Of these, a small number are missing data for one or more of the NHIS variables described below, leaving 72,279 eligible for this study.

### Ambient air monitoring data

The Air Quality System (AQS) database provides annual averages of air monitoring data—ambient concentrations of criteria and hazardous air pollutants at monitoring sites, primarily in cities and towns (U.S. EPA 2008). The AQS database is updated daily by the U.S. EPA, primarily by the staff of state and local environmental agencies that measure ambient concentrations of criteria air pollutants at several thousand monitoring sites throughout the United States. Each month, the U.S. EPA extracts a summary of the measurements recorded at each air monitoring station (e.g., the highest value in a year and the average) and updates its database.

As part of a larger project to create linked NHIS–AQS data files, we extracted monitor-specific annual averages for several pollutants, including O_3_, fine PM (PM with aerodynamic diameter ≤ 2.5 μm; PM_2.5_), respirable PM (PM with aerodynamic diameter ≤ 10 μm; PM_10_), NO_2_, and SO_2_ from the AQS using the annual summary web pages (Parker et al. 2008a); in addition, we calculated exposure to O_3_ during the summer (May through September) for these linked data files using daily measures. We calculated summer O_3_ exposures separately to obtain more comparable measures throughout the United States than annual average O_3_ exposures—although differing monitoring periods are used across geographic areas, summertime is the most frequently monitored period (U.S. EPA 2006).

### Linkage of the NHIS to the U.S. EPA air monitoring data

We assigned pollution exposure values to individual NHIS records for each survey year by averaging the monitor-specific annual averages for each pollutant for that year from monitors within a specified distance, and within the same county, of the respondent. Although the individual observations for each monitor are available for these pollutants, we used the annual arithmetic averages calculated by the U.S. EPA, except for O_3_, as described above. We considered obtaining the annual arithmetic averages from the U.S. EPA preferable to calculating these or other metrics directly from the raw data because they reflect U.S. EPA methodology, are readily obtained, and are more easily replicated (Parker et al. 2008a). We calculated distances between the NHIS participants and the air quality monitors using the latitude and longitude assigned to the block group (population-weighted centroid) of residence of NHIS participants and the latitude and longitude of the U.S. EPA monitors. Using these distances to identify nearby monitors, we calculated annual pollution estimates within specified distances of each NHIS respondent’s block group (Parker et al. 2008a).

For this study, we examined air pollution exposures for PM_2.5_, PM_10_, summer O_3_, SO_2_, and NO_2_ calculated from monitors within a 20-mile radius, averaged using inverse-distance weighting, as the primary exposure measures. We chose these metrics to maximize the number of available children in the analyses. We conducted additional analyses using exposures estimated over 5 miles and with the annual O_3_ exposure available from the U.S. EPA. Although children interviewed at the beginning of the calendar year may be less accurately assigned exposure based on calendar-year estimates because interview questions ask about the previous 12 months, correlations of annual exposures for these pollutants, grouped by county, were nearly always > 0.80 and often > 0.90.

### Variable definitions

We categorized outcomes using responses to the following questions: “During the past 12 months, has [child’s name] had any of the following conditions? Hay fever? Any kind of respiratory allergy? Any kind of food or digestive allergy? Eczema or any kind of skin allergy?” Children whose parents reported either hay fever or respiratory allergy in the previous 12 months were combined into one group because both conditions result in symptoms of allergic rhinitis—watery, itchy eyes, congestion, allergic cough, sneezing, and runny nose. We included children ≥ 3 years of age: Seasonal allergies typically develop after 2 years of age because children must be repeatedly exposed to seasonal pollens before developing a specific immunologic response (Fireman 2000).

We considered several additional factors that could influence observed associations between air pollution and reporting of respiratory allergy/hay fever: survey year, child’s age, family structure (two parents, single mother, other), usual source of care (yes, no), health insurance (insured, uninsured), family income relative to federal poverty level (< 100%, 100% to < 200%, 200% to < 400%, ≥ 400%), child’s race/ethnicity (non-Hispanic black, Mexican or Mexican American, non-Hispanic white, other groups). We separated the Mexican or Mexican-American subgroup from the other Hispanic subgroups because of the heterogeneity among these subgroups (Hajat et al. 2001). For the approximately 30% of records without reported income, we used the NHIS imputed income files to assign family income level (NCHS 2008a). We coded health insurance as insured or uninsured using the definition used by NCHS (2007). These factors are related to the reporting of childhood allergies and may affect estimated associations between allergies and air pollution.

We also included county-level geographic level covariates: median income from the 2000 U.S. Census, an index of urban–rural status (Ingram and Franco 2006), and region, as used by the U.S. EPA (2003). We included urban–rural status and region because components of air pollution may differ geographically. We included median county-level income as a potentially confounding factor, related to both health outcomes (including the reporting of childhood allergies and available health services) and air pollution exposures.

Asthma is an important childhood condition related to the reporting of allergies and air pollution exposures and, in the United States, related to many of the covariates listed above (Akinbami 2006; Johnson et al. 2002). To examine the influence of asthma on possible associations between allergies and air pollution, we defined children as having current asthma if their parent or guardian reported that they had ever been told the child had asthma and that the child had had an asthma attack in the previous 12 months.

Environmental tobacco smoke affects the respiratory health of children. Unfortunately, the NHIS does not collect smoking information from all adults, just from one sampled adult per family. As a result, our smoking information for a child’s exposure can only be dichotomized as yes versus unknown. Data on the smoking status of children is not collected in the NHIS.

### Analysis

We evaluated possible associations between air pollution and childhood respiratory allergy/hay fever using logistic regression models. We used statistical software for surveys (SUDAAN; RTI International 2006) because of the complex clustered sample design of the NHIS. We included the design information, including the strata, PSUs, and adjusted sampling weights, to ensure proper statistical inference.

We fitted unadjusted and adjusted models separately for each pollutant. We tested interaction terms to determine whether associations differed by characteristics described above. We fitted additional models to examine the impact, if any, of current asthma; because the relationship between allergies and asthma among children is complex (Weinmayr et al. 2007), we decided to examine the role of asthma as a potential confounder in this study. However, given that the role of asthma may be more direct, making its inclusion in the models inappropriate, these analyses are secondary to the main analyses. We also examined the effect of the presence of an adult smoker in the family on any observed associations; because this information is not collected for every adult in the household, making the variable imprecise, we included these results in the secondary analyses. Because the subsets of children with each pollution exposure differ, we performed extensive sensitivity analyses, including models with multiple pollutants and with exposures measured over smaller areas.

## Results

All characteristics shown in Table 1 were significantly associated with respiratory allergies/hay fever as demonstrated by chi-square statistics without consideration of multiple comparisons. Using the combined outcome, 19.2% of this cohort of children 3–17 years of age are reported to have had hay fever, respiratory allergy, or both within the previous 12 months (Table 1); of this group, 11.4% reported hay fever, 12.8% reported respiratory allergy, and 5% reported both conditions. These estimates are slightly higher than those reported above (Bloom and Cohen 2007) because of the slightly older study cohort.

The number of children linked to air monitoring data varies by pollutant (Table 1). Of the eligible 72,279 children, 32,137 had exposure data for each of these pollutants within 20 miles, and 47,989 had exposure data for both PM_10_ and PM_2.5_, allowing an approximate estimate of the coarse PM exposure by the difference (PM_2.5–10_ = PM_10_ levels – PM_2.5_ levels). In all linkage groups, respiratory allergy/hay fever reporting was slightly lower than in the overall sample of children, ranging from 18.3% for children linked to NO_2_ to 19.0% for children linked to PM_2.5_. There are small demographic differences and larger geographic differences among the linkage subsets.

Table 2 shows correlations among the pollutants. Generally, correlations are low, with negative correlations between PM_10_ and SO_2_ and between PM_2.5–10_ and SO_2_. Correlations between pollutants were, for the most part, similar whether we used the subset of 32,137 children linked to all pollutants for all calculations or used different subsets, depending on available data, for each calculation.

Associations between reporting of respiratory allergy/hay fever and air pollution varied among the pollutants (Table 3). After adjustment for demographic and geographic variables, increases in summer O_3_ were associated with increased reporting of respiratory allergy/hay fever. PM_2.5_ was associated with reporting of these conditions after including geographic indicators in the models. Other gaseous pollutants were not associated with increased respiratory allergy/hay fever and, in some cases, were negatively associated. After controlling for multiple pollutants (Table 4), associations between reporting of respiratory allergy/hay fever and air pollution persisted for summer O_3_ and PM_2.5_. The association between respiratory allergy/hay fever and PM_2.5–10_ increased in the subset linked to multiple pollutants, both without and with adjustment for multiple pollutants, suggesting possible sample selection effects in this group.

Because including geographic variables had a large impact on the associations, we examined whether effects were similar by urban–rural status by including interaction terms in the regression models. In single-pollutant models, associations were significantly different by urban–rural status for summer O_3_, PM_10_, and PM_2.5–10_, but not for PM_2.5_ or the other gaseous pollutants. Summer O_3_ had a slightly stronger effect on reporting of respiratory allergy/hay fever in the more urban areas compared with the small metropolitan and rural areas, whereas PM_10_ and PM_2.5–10_ had slightly stronger associations in medium and fringe metropolitan areas (data not shown).

Given the differences in reporting respiratory allergy/hay fever by child’s race, family poverty status, and child’s age and sex, we examined interactions (products of these factors and the pollutants) to determine whether associations for summer O_3_ and PM variables differed by these factors. Model results with interaction terms indicated that associations did not differ significantly (using *p* < 0.05 as criterion) by race/ethnicity, sex, or age category for either PM or summer O_3_. Associations did not differ significantly by poverty status for PM variables, but the associations between summer O_3_ exposure and respiratory allergy/hay fever for children in poverty categories > 200% of poverty were stronger than for those < 200% of poverty: <100% poverty, adjusted odds ratio (AOR) = 1.05 [95% confidence interval (CI) = 0.95–1.16]; 100% to < 200%, 1.13 (1.01–1.27); 200% to < 400%, 1.28 (1.17–1.39); 400% or higher, 1.25 (1.15–1.35).

Because the 20-mile exposure radius may be inadequate for assigning exposure for the gaseous pollutants, we replicated the full models using the subsets of children within 5 miles of pollution monitors. Fewer than half of the children with 20-mile exposure variables were available for the 5-mile analyses, ranging from 14,034 for SO_2_ to 26,898 for PM_2.5_. Results for summer O_3_ from a model adjusted for demographic and geographic variables remained statistically significant (OR = 1.16; 95% CI, 1.07–1.25), if slightly weaker than the OR shown in Table 3. With the smaller sample size, results for PM_2.5_ were elevated but lower than those shown in Table 3, and no longer significant (OR = 1.09; 95% CI, 0.93–1.28). Associations between reporting of respiratory allergy/hay fever and the other pollutants were similar to those reported using the 20-mile exposures shown in Table 3. Correlations between the 20-mile and 5-mile pollution variables were high, especially for summer O_3_ and PM_2.5_ (*r* > 0.95); for the other pollutants, correlations between the 20-mile and 5-mile variable were at least 0.85. Although the 20-mile and 5-mile exposure variables, calculated from many of the same monitors, are not independent and hence statistical inferences from these correlations are inappropriate, the correlations give an indication of the similarity of the measures.

We fit models without controlling for survey year, with similar results (data not shown). Including survey year would be considered appropriate for the control of temporal changes that may have affected reporting of respiratory allergies, such as access to health care; on the other hand, year is also correlated with air pollution levels, and including year would be considered overcontrol if, for example, changes in reporting over time were attributed to the corresponding temporal changes in air pollution. Because we cannot distinguish the two scenarios, we examined results both ways.

We compared the results for the summer O_3_ with those using the annual averages of the daily 8-hr running O_3_ averages provided by the U.S. EPA. These averages were, by design, lower than the summer averages but highly correlated (*r* = 0.80) despite the differing O_3_ reporting periods throughout the United States, the use of average daily measurement to calculate the summer-O_3_ variable, and the use of daily 8-hr averages to calculate the annual O_3_ variable. Corresponding to the high correlation, the association between annual O_3_ exposure and respiratory allergy/hay fever adjusted for demographic and geographic factors (AOR = 1.16; 95% CI, 1.11–1.21) was similar to that reported for summer O_3_ (Table 3).

In further tabulations of our study sample, of the children with respiratory allergy/hay fever, > 30% had been told at some time that they had asthma; in contrast, only 9% of children without allergies or hay fever had been told they had asthma. Adjustment for asthma in the logistic regression models did not change the findings for either summer O_3_ or PM_2.5_, although the independent effect of asthma on respiratory allergy/hay fever was large (data not shown).

Finally, we fit models that included adult smoking (yes vs. unknown). In the eligible sample, 20.3% of children lived in a family with a known smoker. Although adult smoking in the family had an independent effect on respiratory allergy/hay fever, its inclusion in the models did not affect associations between respiratory allergy/hay fever and either PM_2.5_ or O_3_ (data not shown).

## Discussion

We found a persistent association between increased levels of summer O_3_ and reporting of respiratory allergies and hay fever among U.S. children. These findings were stronger in urban than in less urban areas and persisted when controlling for family income, parental education, family structure, child’s age, median county-level income, access to care, and current asthma status. The association between O_3_ and respiratory allergy/hay fever was weaker among children living in the less metropolitan counties, which may suggest variation in types of allergens or other factors related to level of urbanization (climate, societal) that modify the effects of O_3_ on respiratory allergies/hay fever. Furthermore, that the association became stronger as family income rose suggests one of two scenarios: *a*) Real differences exist and there is something about more privileged children that increases their susceptibility; or *b*), parents in lower-income groups are underreporting.

Although the findings for increased reporting of respiratory allergy/hay fever with increased O_3_ exposure were most robust, we also found a strong association between PM_2.5_ exposure and reporting of respiratory allergy/hay fever after controlling for geographic variables. Effects of PM_2.5–10_ on the reporting of respiratory allergy/hay fever were weaker than the effects of PM_2.5_ and varied somewhat geographically.

In contrast, we found no evidence with these data that annual average exposure to NO_2_ or SO_2_ when derived by spatial averages within 20 or within 5 miles of a child’s residence was associated with reporting of childhood respiratory allergy/hay fever. Although these findings may indeed reflect no association, there is some evidence that metrics for these gaseous pollutants based on ambient monitoring are less representative of exposure than are similar metrics for O_3_ or PM (Ito et al. 2005; Kim et al. 2006).

Our findings for O_3_ are consistent with a recent study of > 6,000 children in France that showed increased allergic sensitivity and lifetime allergic rhinitis with increasing O_3_ exposure (Pénard-Morand et al. 2005) and a time series study from England that showed physician visits for allergies increased with increasing O_3_ exposure among children (Hajat et al. 2001); in addition, a study of Austrian school children (Frischer et al. 2001) showed increases in a biomarker related to airway inflammation (urinary eosinophil protein X) and respiratory response with increasing O_3_ exposure. However, studies from France (Ramadour et al. 2000) and Taiwan (Hwang et al. 2006) showed no association between O_3_ and measures of respiratory allergy. In the United States, earlier results from the Harvard Six Cities study showed an elevated, but not statistically significant, risk of hay fever in cities with higher O_3_ levels (Dockery et al. 1989). A study of 170 German school children showed that the increased allergic responses associated with increased levels of O_3_ were reduced with constant high levels of O_3_, possibly indicating an adaptive response (Kopp et al. 1999). How this potentially adaptive response affects overall associations in a study area as large as the United States with widely varying underlying O_3_ levels is unknown.

Fewer studies have specifically examined PM. Recent German studies of PM_2.5_ found increased allergic symptoms (sneezing/runny nose) for very young children (Morgenstern et al. 2007) and allergic sensitization and hay fever for these children up to 6 years of age (Morgenstern et al. 2008) exposed to higher pollution levels. In a study in Paris, Nikasinovic et al. (2006) observed associations between markers of nasal inflammation and PM_2.5_ exposure in children with mild-to-moderate allergic asthma but not in other children. Similarly, a separate study in France showed a relatively large effect of PM_2.5_ on allergic asthma but not on allergic rhinitis (Annesi-Maesano et al. 2007). The studies by Pénard-Morand et al. (2005) and Hajat et al. (2001), mentioned above, also showed positive associations between high PM_10_ levels and lifetime allergies and doctor visits, respectively. On the other hand, a study of schoolchildren in Norway generally showed no associations between traffic-related pollutants, including PM_2.5_, and allergen sensitization (Oftedal et al. 2007); the authors suggested that Oslo pollution levels are too low to detect associations. Similarly, in a prospective cohort study of children from the Netherlands, PM_2.5_ was not associated with allergic sensitization, except for food allergies (Brauer et al. 2007).

Inferences for the effects of PM on allergies, for the most part, have been based on traffic studies. Studies from Germany have arrived at different conclusions. One German study showed elevated but not significantly elevated prevalence of hay fever for children exposed to more traffic; in this study, traffic-related pollution was associated with allergic sensitization among children who were also exposed to tobacco smoke (Nicolai et al. 2003). Other German studies showed increased hay fever and other allergic symptoms with markers of traffic exposure (Duhme et al. 1996; Krämer et al. 2000; Weiland et al. 1994); however, another study from Germany showed no associations between traffic indices and allergic symptoms (Hirsch et al. 1999). A study of Dutch children related truck, but not automobile, traffic to increased respiratory symptoms and allergy sensitization (Janssen et al. 2003); an earlier Dutch study showed elevated but not significantly higher risks of allergies for children living on busy streets compared with those living on quieter streets (Oosterlee et al. 1996). From Asia, studies from Thailand (Pothikamjorn et al. 2002) and Taiwan (Lee et al. 2003) showed more allergic symptoms for children exposed to more traffic. Although differences among these studies can readily be attributed to differences in measures of traffic exposure and markers of allergy, the collection supports our finding that increases in PM exposure can increase the burden of allergies among children.

Prior summaries of the epidemiology of respiratory allergies and hay fever are often coupled with examinations of other respiratory symptoms, especially asthma (Brunekreef and Sunyer 2003; Johnson et al. 2002; Nicolai 2002). Most children with asthma also report respiratory allergies/hay fever; both diseases cover a spectrum of conditions and can be triggered by similar agents (e.g., pollen or cat). Although a detailed examination of air pollution effects on indicators of asthma was not part of this study, our results for effects of O_3_ and PM_2.5_ on reported allergy persisted after controlling for current asthma. More targeted analysis is required to understand the effects of air pollution on the co-reporting of respiratory allergies/hay fever and indices of asthma.

The strength of this study is its nationwide scope and its large number and diversity of children. The NHIS is carefully conducted, and survey weights are created to reflect all children in the United States. These data contain information on family characteristics, including family income and education of family members, to control for confounding. Using in-house geographic information, we could additionally consider geographic factors. Even with the reduced sample available to examine air pollution, the survey weights allow estimates to better reflect the population of children living near monitors throughout the United States.

A limitation of this study is its cross-sectional design, intended to provide prevalence estimates. From this design, we cannot establish the direction of the air pollution—hay fever/respiratory allergy association, and our results may be subject to length bias, where children with longer duration of hay fever/respiratory allergy will overrepresent cases compared with children with shorter duration (Rothman et al. 2008). Additionally, the definition of the primary outcome variable by parent self-report and the 12-month time span for the recall may lead to additional bias. With this case definition, we cannot, for example, examine the role of the timing of air pollution on the initiation of either atopic sensitization or the onset of symptoms. As expressed by Johnson et al. (2002), defining allergies is difficult—“the most significant and as yet unsolved methodological issue is one fundamental to the conduct of epidemiology: arriving at a definition of disease.” Commonly used biomarkers for allergy— positive blood tests for allergen-specific serum immunoglobulin E and positive allergen-specific skin prick tests—are not always correlated with each other or with clinical allergic symptoms (Johnson et al. 2002). Allergies are also characterized by allergic sensitization measures, although correlates of respiratory allergic symptoms are not necessarily the same as those for sensitization. A generally used definition of childhood allergies is based on parental report of symptoms or report of a history of a provider diagnosis, similar to the case definition based on parental report in this study. As a consequence, making inferences from studies using different case definitions about allergy prevalence, onset, severity, or exacerbation is difficult.

We used a data set previously linked to annual exposure data (or summer-O_3_ data), which may not cover the exact recall period for each respondent. These annual exposures, however, are correlated such that exposure for the calendar year is probably similar to exposure for the previous year; averaged over counties, the correlations between adjacent average exposures were high (e.g., for summer O_3_, *r* = 0.85; for PM_2.5_, *r* = 0.80). In addition, we have no information on local aeroallergen levels, which would have increased our understanding of the effects of air pollution on reported allergies.

Another limitation is the primary use of a 20-mile radius to define exposure from the NHIS respondent’s residential block group. Ideally, more precise exposure measures would be available for each child, either from personal monitoring or finer spatial scales, which would lead to more precise effect estimates. With this survey, there is a substantial trade-off between criteria for proximity of monitors and the sample size and composition of the available data (Parker et al. 2008a, 2008b). In this case, the results using the substantially smaller samples within 5 miles of a pollution monitor were remarkably similar to the primary results presented. This similarity was apparent for both the PM exposures and the gaseous pollutant exposures. Indeed, this similarly suggests a robustness of the findings to both sample composition and exposure assignment. With the national data, spatial variation in exposure is relatively large; as evidenced by the correlations, rankings of the pollutant values are similar within the sample whether defined by 20 miles or 5 miles. Markedly few of these children lived within 1 mile of an O_3_ (< 500) or PM_2.5_ (< 3,000) monitor and were likely poorly distributed demographically and geographically across the United States.

A related issue is our impaired ability to compare within-area effects of local differences in exposure with between-area effects. The NHIS is not designed for local estimates; although it may be possible to define a local unit of analysis from which to frame the comparison, say, county or metropolitan statistical area, the clustered design does not lend itself to sufficiently varied exposures within most counties to adequately examine this question.

Children may be especially sensitive to effects of inhaled toxins and pollutants. Compared with adults, they have higher metabolic rates and minute ventilation rates (Bateson and Schwartz 2008). Most important, lung growth development continues through childhood and may be altered by environmental exposures. Studies of infant nonhuman primates exposed to O_3_ for cyclical periods over 6 months demonstrated altered airway architecture with fewer and narrower small airways, disordered smooth muscle orientation, and hyperplastic bronchiolar epithelium (Fanucchi et al. 2006). In a prospective study of California children 10–18 years of age, living within 500 m of a freeway had an adverse effect on lung growth (Gauderman et al. 2007). In addition to lung development, sensitivity to allergens may also be affected by pollutant exposure over time. Exposing infant nonhuman primates to both O_3_ and house dust mite allergen showed that O_3_ had a synergistic effect on the effects of allergen exposure on atypical development of the basement membrane zone of the airway epithelium and alterations in immunoreactivity (Evans et al. 2003). Although the O_3_ level in these studies (0.5 ppm) exceeded the average levels found in our study, these studies present evidence of permanently compromised lung structure and growth for early or chronic exposure to pollutants.

There have been several studies of children’s respiratory health and air pollution from the United States. Although these studies focused more on allergies and other respiratory symptoms, they highlight the importance of air pollution for children’s health. For example, two multisite studies primarily related to asthma, the National Cooperative Inner-City Asthma Study (Mortimer et al. 2002) and a study of children in the Childhood Asthma Management Program (Schildcrout et al. 2006), reported increased asthma symptoms with increasing air pollutants. Poorer lung function was related to PM exposure among asthmatic children from Detroit, Michigan (Lewis et al. 2005). Similarly, Gent et al. (2003) reported exacerbation of respiratory systems with increasing levels of O_3_ and PM_2.5_ among asthmatic children in New England. The Children’s Health Study from Southern California (Peters et al. 1999) has reported increased asthma medication use and wheezing (Millstein et al. 2004), school absenteeism (Gilliland et al. 2001), and poorer lung development (Gauderman et al. 2004) among children exposed to different air pollutants. Using data from East Bay Children’s Respiratory Health Study, Kim et al. (2004) found that traffic-related pollution significantly increased respiratory symptoms among children in Northern California. Earlier studies of respiratory illness among children from the Harvard Six Cities Study (Dockery et al. 1989) and among 24 communities throughout the United States and Canada (Dockery et al. 1996) reported increases in some but not all symptoms and measures of PM.

## Conclusion

The results of this study agree with the findings in the literature: exposures to O_3_ and fine PM are associated with childhood respiratory allergies. The weight of the findings to date support detailed monitoring of air quality as high levels of exposure are increasingly being demonstrated as unsafe to public health and costly in terms of morbidity, lost school and work days, and lower quality of life.

## Figures and Tables

**Table 1:** Characteristics of eligible study population, percentage of children with respiratory allergies/hay fever, and characteristics of children linked to air pollution.

			Characteristics of children by linkage to each pollutant (% distribution)				
Characteristic	All eligible children (% distribution) (*n* = 72,279)	Respiratory allergy/hay fever (%)^a^	Summer O_3_ (*n* = 58,147)	SO_2_ (*n* = 42,791)	NO_2_ (*n* = 42,467)	PM_2.5_ (*n* = 57,273)	PM_10_ (*n* = 50,874)
All	100.0	19.2	100.0	100.0	100.0	100.0	100.0
Percent of poverty threshold							
< 100	17.3	15.4	16.6	16.9	17.5	16.9	17.4
100 to < 200	21.5	17.1	20.4	20.2	20.5	20.7	21.0
200 to < 400	32.5	19.7	31.9	31.5	30.4	31.7	31.3
≥ 400	28.7	22.5	31.1	31.4	31.6	30.7	30.3
Insurance coverage							
Yes	89.4	19.8	89.7	89.8	88.9	89.7	89.2
No	10.6	13.8	10.3	10.2	11.1	10.3	10.8
Usual source of care							
Yes	94.1	19.7	94.4	94.6	93.9	94.3	93.9
No	5.9	11.1	5.6	5.4	6.1	5.7	6.1
Race/ethnicity							
Non-Hispanic black	14.6	16.8	15.7	17.8	17.5	16.2	16.7
Mexican or Mexican American	11.7	12.6	12.5	11.7	15.3	12.1	14.3
Non-Hispanic white	62.4	21.4	59.0	56.6	51.8	59.0	55.3
All other groups	11.3	16.7	12.8	13.9	15.4	12.8	13.6
Highest level of education of adult in household							
Less than high school	11.3	11.8	11.6	12.0	13.1	11.6	12.5
High school graduate	23.5	16.3	22.2	22.1	21.4	22.1	21.6
Some college	33.1	21.0	32.2	31.6	31.2	32.3	32.2
College graduate	19.7	21.5	20.6	20.6	20.6	20.7	20.5
Postgraduate	12.4	23.0	13.4	13.8	13.7	13.4	12.2
Family structure							
Two parents	61.0	19.5	60.0	58.7	57.8	60.0	58.9
One parent	15.9	19.6	16.1	16.8	16.5	16.3	16.6
Other groups	23.1	18.0	23.8	24.5	25.7	23.7	24.5
Age (years)							
3–5	19.6	14.3	19.9	20.0	20.1	20.1	20.0
6–9	26.3	18.1	26.4	26.5	26.4	26.4	26.4
10–14	34.1	21.1	34.0	34.1	34.0	34.1	34.0
15–17	20.0	22.1	19.8	19.5	19.5	19.4	19.6
Level of urbanization							
Large central metro	28.1	16.6	35.2	42.5	47.6	34.5	40.6
Large fringe metro	25.1	20.4	29.5	31.7	32.4	28.2	27.8
Medium metro	20.1	20.3	23.3	18.8	16.4	22.9	21.1
Small metro and other areas	26.0	19.9	12.0	7.1	3.7	14.4	10.5
Region							
Northeast	21.4	18.8	24.9	30.1	27.9	23.2	21.7
Southeast	26.1	20.3	24.4	21.8	22.2	25.0	24.1
Industrial Midwest	21.5	19.3	21.1	22.4	16.6	20.6	19.0
Upper Midwest	6.9	18.5	4.9	5.0	3.9	5.3	4.9
Southwest	5.6	20.8	4.7	3.9	6.1	5.0	5.7
Northwest	10.9	21.5	10.6	7.1	10.2	11.9	13.8
Southern California	7.6	12.3	9.3	9.7	13.2	9.0	10.9

All percentages were weighted using NHIS survey weights.

a All *p* < 0.05 for associations between variables and respiratory allergy/hay fever.

**Table 2:** Median (interquartile range) and correlations among pollution variables within a 20-mile radius of study subjects.^a^

	Pollutant					
Measure	Summer O_3_ (ppb)	SO_2_ (ppb)	NO_2_ (ppb)	PM_2.5_ (μg/m^3^)	PM_2.5–10_ (μg/m^3^)	PM_10_ (μg/m^3^)
Median (interquartile range)	31.5 (27.6–35.1)	3.90 (2.35–5.50)	17.8 (13.6–23.4)	13.1 (10.9–15.2)	11.2 (8.2–15.2)	24.1 (20.8–28.7)
Correlation						
Summer O_3_	1.00	0.00	−0.07	0.10	0.16	0.26
SO_2_		1.00	−0.26	0.21	−0.33	−0.19
NO_2_			1.00	0.53	0.29	0.48
PM_2.5_				1.00	0.02	0.51
PM_2.5–10_					1.00	0.86
PM_10_						1.00

a Number of children with pollutant exposure is given in Table 1. Number of children with data for two pollutants varies by combination.

**Table 3:** ORs (95% CIs) per approximate interquartile range^a^ for associations between annual average air pollution exposures and reporting of respiratory allergy/hay fever among children.

		Single-pollutant models		
Pollutant	No. of children	Unadjusted	Adjusted^b^	Adjusted^c^
Summer O_3_	58,147	1.18 (1.13–1.23)	1.15 (1.11–1.20)	1.20 (1.15–1.26)
SO_2_	42,791	1.05 (1.01–1.09)	1.00 (0.96–1.04)	1.03 (0.97–1.08)
NO_2_	42,467	0.83 (0.79–0.86)	0.87 (0.84–0.92)	0.95 (0.90–1.01)
PM_2.5_	57,273	0.79 (0.74–0.84)	0.87 (0.80–0.93)	1.16 (1.04–1.30)
PM_2.5–10_	47,867	0.92 (0.89–0.97)	1.02 (0.97–1.06)	1.01 (0.95–1.07)
PM_10_	50,874	0.89 (0.86–0.92)	0.97 (0.94–1.01)	1.04 (0.99–1.09)

We averaged O_3_ for May through September; all other pollutants represent annual averages.

a Summer O_3_ per 10 ppb; SO_2_ per 3 ppb; NO_2_ per 10 ppb; PM per 10 μg/m^3^.

b Adjusted for year, poverty, race, family structure, insurance, usual source of care, age, and education of adult.

c Additionally adjusted for urban status, region, and median income of county.

**Table 4:** AORs (95% CIs) per approximate interquartile range^a^ for associations between annual average air pollution exposures and reporting of respiratory allergy/hay fever among children: single- and multiple-pollutant models (*n* = 32,080).

Pollutant	Single-pollutant model	Multiple-pollutant model
Summer O_3_	1.24 (1.15–1.34)	1.18 (1.09–1.27)
SO_2_	0.95 (0.88–1.02)	0.97 (0.90–1.04)
NO_2_	0.98 (0.90–1.07)	0.99 (0.89–1.10)
PM_2.5_	1.23 (1.04–1.46)	1.29 (1.07–1.56)
PM_2.5–10_	1.13 (1.05–1.22)	1.16 (1.06–1.24)

We averaged O_3_ for May through September; all other pollutants represent annual averages. Adjustments include year, poverty, race, family structure, insurance, usual source of care, age, education of adult, urban status, region, and median income of county. The multiple-pollutant model includes each pollutant; the single-pollutant models are separate models for each pollutant, using the same children as the multiple-pollutant model.

a Summer O_3_ per 10 ppb; SO_2_ per 3 ppb; NO_2_ per 10 ppb; PM per 10 μg/m^3^.
